# Evidence for Widespread Associations between Neotropical Hymenopteran Insects and Actinobacteria

**DOI:** 10.3389/fmicb.2017.02016

**Published:** 2017-10-17

**Authors:** Bernal Matarrita-Carranza, Rolando D. Moreira-Soto, Catalina Murillo-Cruz, Marielos Mora, Cameron R. Currie, Adrián A. Pinto-Tomas

**Affiliations:** ^1^La Selva Biological Station, Organization for Tropical Studies, Heredia, Costa Rica; ^2^Centro de Investigación en Estructuras Microscópicas, Universidad de Costa Rica, San José, Costa Rica; ^3^Centro de Investigación en Enfermedades Tropicales, Universidad de Costa Rica, San José, Costa Rica; ^4^Centro de Investigación en Biología Celular y Molecular, Universidad de Costa Rica, San José, Costa Rica; ^5^Department of Bacteriology, University of Wisconsin-Madison, Madison, WI, United States; ^6^Departamento de Bioquímica, Facultad de Medicina, Universidad de Costa Rica, San José, Costa Rica

**Keywords:** symbiosis, ants, bees, wasps, *Streptomyces*, *Cordyceps*, *Ophiocordyceps*, *Hirsutella*

## Abstract

The evolutionary success of hymenopteran insects has been associated with complex physiological and behavioral defense mechanisms against pathogens and parasites. Among these strategies are symbiotic associations between Hymenoptera and antibiotic-producing Actinobacteria, which provide protection to insect hosts. Herein, we examine associations between culturable Actinobacteria and 29 species of tropical hymenopteran insects that span five families, including Apidae (bees), Vespidae (wasps), and Formicidae (ants). In total, 197 Actinobacteria isolates were obtained from 22 of the 29 different insect species sampled. Through 16S rRNA gene sequences of 161 isolates, we show that 91% of the symbionts correspond to members of the genus *Streptomyces* with less common isolates belonging to *Pseudonocardia* and *Amycolatopsis*. Electron microscopy revealed the presence of filamentous bacteria with *Streptomyces* morphology in brood chambers of two different species of the eusocial wasps. Four fungal strains in the family Ophiocordycipitacea (Hypocreales) known to be specialized insect parasites were also isolated. Bioassay challenges between the Actinobacteria and their possible targeted pathogenic antagonist (both obtained from the same insect at the genus or species level) provide evidence that different Actinobacteria isolates produced antifungal activity, supporting the hypothesis of a defensive association between the insects and these microbe species. Finally, phylogenetic analysis of 16S rRNA and *gyrB* demonstrate the presence of five *Streptomyces* lineages associated with a broad range of insect species. Particularly our Clade I is of much interest as it is composed of one 16S rRNA phylotype repeatedly isolated from different insect groups in our sample. This phylotype corresponds to a previously described lineage of host-associated *Streptomyces*. These results suggest *Streptomyces* Clade I is a Hymenoptera host-associated lineage spanning several new insect taxa and ranging from the American temperate to the Neotropical region. Our work thus provides important insights into the widespread distribution of Actinobacteria and hymenopteran insects associations, while also pointing at novel resources that could be targeted for the discovery of active natural products with great potential in medical and biotechnological applications.

## Introduction

Hymenoptera is the third largest species-rich insect order, containing more than 150000 extant species described to date (Aguiar et al., 2013), only surpassed by Coleoptera and Lepidoptera. All ants, bees, and wasps belong to this order and, together with termites (Blattodea), represent the best-known groups of insects where eusociality, the highest-level of social organization, is present. Eusociality is characterized by the presence of reproductive division of labor, brood care and overlapping of generation of individuals of the same colony (Wilson, 1971). These complex social systems and their associated adaptations are what have allowed some hymenopteran groups to achieve ecological dominance playing prominent roles in ecosystem function as pollinators (stingless bees and the honey-bee), herbivores (leaf-cutter ants) and predators (e.g., army ants and paper wasps; Hanson and Nishida, 2016). The success of eusocial insects is reflected for example in tropical forests where they can represent more than 50% of all animal biomass (Hölldobler and Wilson, 2009).

However, social lifestyles also represent an opportunity for pathogens and parasites to exploit means of dispersion between colony members in order to infect healthy individuals. Moreover, environmental conditions such as humidity and stable temperatures inside the nest are factors that predispose these insects to pathogenic infestations (Oi and Pereira, 1993).

As defensive responses against pathogenic microorganisms, many social Hymenoptera have developed collective hygienic strategies and altruistic behaviors in order to evade, control or eliminate parasitic infections (Wilson-Rich et al., 2009). Beneficial microbial associations may augment integral immune defenses and help provide protection against pathogens via microbial competition, by stimulating immune responses, or through secretion of anti-microbial compounds (Berg, 1996; Bot et al., 2002; Dillon and Dillon, 2004; Evans and Lopez, 2004; de Souza et al., 2009; Kaltenpoth and Engl, 2013).

As suggested by Kaltenpoth (2009), the factors that may have predisposed Actinobacteria to engaging in defensive symbiotic associations with insects are their ubiquity in terrestrial environments (Mayfield et al., 1972; van der Meij et al., 2017), the capability to degrade recalcitrant carbon and nitrogen sources (e.g., lignin, chitin and cellulose; Bhattacharya et al., 2007; Větrovský et al., 2014), and the potential to produce many bioactive secondary metabolites (Barka et al., 2016). The genus *Streptomyces* alone produces more than 80% of all antibiotics with pharmaceutical applications that are used today, either directly as natural products or their semisynthetic derivatives (Bérdy, 2005).

There are two well-studied hymenopteran groups that have established defensive symbiotic associations with Actinobacteria. The first is comprised of leaf-cutter ants and bacteria in the genus *Pseudonocardia.* These bacteria are found on the cuticle of the ants and can occur in crypts in their exoskeleton (Currie et al., 2006), and they produce secondary metabolites that can inhibit the growth of parasitic fungi (*Escovopsis*) that can colonize the ant’s fungal cultivar (Currie et al., 1999; Oh et al., 2009; Marsh et al., 2013; Sit et al., 2015). The second group consists of solitary wasps belonging to the genera *Philanthus*, *Trachyphus*, and *Philanthinus* (Kaltenpoth et al., 2005, 2010, 2012) in which females harbor *Streptomyces* bacteria in specialized gland reservoirs inside the antenna. Before laying their eggs, female wasps secrete their *Streptomyces* symbiont inside the brood chambers. During larval development, the *Streptomyces* produce a mixture of at least nine different antimicrobial compounds that will cover immature wasps and protect them from fungal attack during hibernation periods (Kaltenpoth et al., 2005; Kroiss et al., 2010). Actinobacteria have been characterized in several other social Hymenoptera, including within three ant-plant systems (Hanshew et al., 2015) and in bees (Promnuan et al., 2009), suggesting these associations may be more widespread.

Neotropical Hymenoptera are an extremely diverse group of insects (Hanson and Gauld, 1995) with largely unexplored Actinobacteria associations. Here we examine possible associations between Actinobacteria and tropical eusocial and subsocial Hymenoptera, including species of bees, wasps, and ants, in a tropical rain forest at La Selva Biological Station in Costa Rica (Bawa et al., 1994). Culture-dependent targeted isolations for Actinobacteria from 29 hymenopteran species were conducted. Patterns of associations between insect species and Actinobacteria isolates were characterized through 16S rRNA and *gyrB* DNA sequencing and Bayesian phylogenetic analyses. Moreover, different strains of entomopathogenic fungi known to infect some of the insects under study, including strains in the genus *Ophiocordyceps* (*Hirsutella*) were isolated. These fungi are known as highly specialized pathogens capable of manipulating the behavior of their host (de Bekker et al., 2014). Finally, the results from *in vitro* bioassays show that some strains of Actinobacteria associated with different species of insects were capable of inhibiting the growth of these entomopathogenic fungi.

## Materials and Methods

### Insect Sampling for Actinobacteria Screening

Insect colonies were collected at La Selva Biological Station (Organization for Tropical Studies) from 2010 to 2014 (**Table 1**). This station is located in the province of Heredia, Costa Rica (10° 25′ 53.14″ N, 84° 0′ 10.51″ W) and is composed of 1500 hectares of lowland tropical rain forest. A few other samples were collected in Las Brisas Nature Reserve, Limón Province, Costa Rica (10° 04′ 09.7″ N, 83°37′ 57.5″ W). Samples from each colony were aseptically collected using flame sterilized forceps and placed directly into a capped sterile container for transport to the laboratory. Each colony was divided into different sub-samples for further Actinobacteria isolation. Colony components when present included larvae, adults, nest material and honey or pollen in the case of bees. Our sampling approach encompassed eusocial insects (all ants, paper wasps, and stingless bees) and non-eusocial insects (bees from the tribe Euglossini, and wasps from the family Pompilidae and Crabronidae).

**Table 1:** Insect species and colony components sampled and Actinobacteria isolates obtained.

			Number of isolates obtained from:				
Insect family	Species	Colonies sampled	Adult	Larvae	Nest	Others	Total
Apidae	*Euglossa allosticta*^‡^	1	0	—	—	—	0
	*Euglossa hansoni*^‡^	1	0	—	—	—	0
	*Euglossa heterosticta*^‡^	6	2	—	—	—	2
	*Euglossa ignita*^‡^	1	0	—	—	—	0
	*Euglossa imperilais*^‡^	2	2	—	—	—	2
	*Euglossa cybelia*^‡^	1	0	0	0	—	0
	*Eulaema speciosa*^‡^	2	0	0	0	0	0
	*Tetragonisca angustula*^†^	16	7	2	0	10^∗^	19
	*Trigona* sp. 1 ^†^	4	0	—	0	—	0
Formicidae ^†^	*Crematogaster longispina*	10	5	4	1	—	10
	*Odontomachus bauri*	3	0	1	0	—	1
	*Odontomachus erythrocephalus*	18	11	7	3	—	21
	*Odontomachus hastatus*	1	0	1	0	—	1
	*Odontomachus opaciventris*	5	0	2	0	—	2
	*Paraponera clavata*	16	18	—	—	—	18
	*Paratrechina caeciliae*	10	7	4	1	—	12
	*Pheidole bicornis*	13	0	2	5	—	7
	*Pheidole fiorii*	8	4	1	0	—	5
	*Tapinoma ramulorum inrectum*	8	4	1	3	—	8
Pompilidae ^‡^	Pompilidae sp. A	3	1	2	0	—	3
Crabronidae ^‡^	*Trypoxylon* sp. A	4	3	8	4	—	15
	*Trypoxylon* sp. B	1	1	1	0	—	2
Vespidae ^†^	*Agelaia cajennensis*	11	3	16	3	—	22
	*Metapolybia docilis*	10	3	6	15	—	24
	*Polybia plebeja*	13	1	6	5	—	12
	*Polybia occidentalis bohemani*	7	2	2	4	—	8
	Vespidae sp. A	1	0	0	0	—	0
	Vespidae sp. B	1	0	1	0	—	1
	Vespidae sp. C	1	0	1	1	—	2
Total		178	74	68	45	10	197


^†^*Eusocial insects studied (all ants, paper wasps, and stingless bees). ^‡^Non-eusocial insects (mud dauber wasps and Euglossini bees). ^∗^Isolates obtained as follows: three from honey, one comb, one pollen, and five propolis*.

Ten different species of ants representing four sub-families and spanning different lifestyles and habitats were collected. The ant species sampled comprised, *Tapinoma ramulorum* (Dolichoderinae), *Paratrechina caeciliae* (Formicinae), and *Pheidole fiorii* (Myrmicinae), which build carton nests on the leaves of different plants, and *Pheidole bicornis* (Myrmicinae) that obligatorily lives in cavities of the petioles and stems shrubs from the genus *Piper.* Four different ant species in the genus *Odontomachus* (Ponerinae) which live under rocks or trunks, were also sampled. Finally, *Paraponera clavata* (Paraponerinae), an omnivorous large ant that constructs nests in the soil next to the base of trees and is a member of a recent paraphyletic ancestor group to the formicoid ants lineage was also studied (Ward, 2014).

In the case of eusocial wasps, Actinobacteria associations with colonies of *Agelaia cajennensis*, *Polybia plebeja*, *Polybia occidentalis*, and *Metapolybia docilis* were studied. Subsocial wasps in the families Pompilidae and Crabronidae were also sampled (**Table 1**). The Crabronidae family is related to the Apidae lineage (Debevec et al., 2012) and known as mud daubers together with Sphecidae.

From the eusocial stingless bees, *Tetragonisca angustula* and *Trigona* sp. 1 were investigated. Adults, larvae, comb, honey, and pollen from colonies of *T. angustula* were collected; antimicrobial activity has been reported for this species’ honey (DeMera and Angert, 2004). Adults and beehive entrance material were the only colony components of the *Trigona* sp. 1 collected.

Five species of the weakly social orchid bees (Cardinal et al., 2011) were studied using essential oils as bait to collect males (**Table 1**). Finally, adults and larvae specimens from two colonies of the solitary-nesting orchid bee *Eulaema speciosa* were included in the study.

### Actinobacteria Isolation

A culture-dependent approach for isolating Actinobacteria was performed, using chitin agar as a selective media (Cafaro and Currie, 2005). For small insects (e.g., *C. longispina, T. ramulorum, P. bicornis, P. fiorii*, and *P. caeciliae*) five adults, five immature forms (larvae and pupae) and five nest fragments from each colony were placed separately into 0.5 mL of 0.1% sterile tween-20 PBS buffer solution in an autoclaved 1.5 mL Eppendorf tube. For medium sized insects (*T. angustula, Odontomachus* ants, and all wasps), pools of three adult insects or larvae were used. For *P. clavata* ants, only adult insects were collected for Actinobacteria isolation. Each sample was vortexed for 15–20 s. Tween-20 PBS solution was pipetted off, discarded and replaced with 0.5 mL of PBS solution. The sample was macerated with a sterile pestle and 0.5 mL of PBS was added. Then the microcentrifuge tube was vortexed for 15 s. An aliquot of 100 μL was plated onto chitin agar plates and incubated at 27°C for three to 4 weeks. Isolated Actinobacteria were subcultured onto yeast-malt extract agar (YMEA) with antifungals (nystatin and cycloheximide) for macro and micromorphology examination. A Gram stain screening of the isolates was performed prior to DNA extractions of the most promising isolates. A 20% glycerol stock of Actinobacteria spores and cells were prepared by freezing a homogenized mixture in liquid nitrogen and storing them in cryovials at -80°C.

### Entomopathogenic Fungi Sampling and Isolation

A total of 14 insect cadavers with conspicuous signs of *Cordyceps*-like fungal infection were collected and brought to the lab for processing. The majority (11) of specimens were collected in the same geographic area where insect colonies for Actinobacteria screening were sampled. Two wasps (*Agelaia areata* and *Polybia* sp.) and one *Camponotus* ant cadavers were collected in a secondary forest in San Carlos, Costa Rica (10° 34′ 26.2″ N, 84°30′ 08.2″ W). Insect cadavers corresponding to the same species or genus as one of the Actinobacteria hosts under study were chosen for fungal isolation, further characterization and bioassay analysis.

*Ophiocordyceps* infected specimens were of much interest as these fungi have been described as being a hymenopteran host specialist having a narrow host range (Boomsma et al., 2014). Specimens were carefully collected, including a portion of the substrate (leaves or twigs) at which they were attached to avoid damaging the specimen. Samples were placed in sterile plastic containers and brought to the laboratory for processing and identification.

Insect cadavers with fruiting bodies developed or with active conidiophores on stromata were attached with double-sided tape to the inner side of a Petri plate lid. Then the lid was placed on a plate containing PDA media for collecting discharged ascospores or conidia (Lacey, 2012). Pieces of stromata or synnemata were also cut with sterile scissors and directly stroked against PDA media with flame-sterilized forceps, in a laminar flow hood. Agar plates were examined under a microscope and pure cultures were obtained after cutting pieces of agar with single ascospores or hyphal tips and transferred into new PDA plates that were later incubated at 27°C for 4 weeks. Agar slants with sterile mineral oil and 20% glycerol stocks were prepared to preserve the isolates.

### DNA Extractions and PCR

From each colony component sampled, unique isolate morphotypes were chosen for DNA extraction and 16S rRNA sequencing. Standard cetyltrimethylammonium bromide (CTAB) DNA extractions were performed for Actinobacteria isolates (Cafaro et al., 2011). PCR amplifications were employed on the genomic DNA using the eubacterial universal primers, 27F and 1492R (Lane, 1991) for 16S rRNA. As 16S rRNA gene sequence analysis cannot distinguish between closely related taxa (Han et al., 2012) the *gyrB* coding gene for 71 representative *Streptomyces* isolates from our collection were sequenced, using the primers *gyrB*-F1 (GAGGTCGTGCTGACCGTGCTGCA) and *gyrB*-R1 (GTTGATGTGCTGGCCGTCGACGT) (Hatano et al., 2003). The PCR amplification reactions were performed in a Veriti (Applied Biosystems) Thermal cycler. The reaction conditions for 16S rRNA gene amplification consisted of 35 cycles at 94°C for 1 min, 52°C for 1 min, 72°C for 1 min. The amplification of *gyrB* consisted of 30 cycles of denaturation at 95°C for 1 min, annealing for 0.5 min at 65°C, and extension at 72°C for 1.5 min. Bacterial 16S rRNA and *gyrB* sequences obtained in this study were deposited in the GenBank database under accession numbers KY067229-KY067322 and KY082974-KY083044, respectively.

In the case of fugal genomic DNA extractions, a simple thermolysis method was applied (Zhang et al., 2010). Mycelium from 3-day cultures from fast growing isolates was used to extract DNA. In the case of *Ophiocordyceps* isolates PCFB and BA18 fresh stromata tip from old cultures were used. PCR amplification of ITS region was performed with primers ITS4 and ITS5 (White et al., 1990). SSU was amplified using the primers NS1 and NS4 (White et al., 1990). Amplification of the elongation factor 1-α (EF1-α) was performed with the primers 983F and 2218R and cycling conditions used by Rehner and Buckley (2005). ITS amplification cycling conditions were of 35 cycles of 0.5 min at 95°C, 0.5 min at 52°C and 1.5 min at 72°C. SSU amplification was performed with 35 cycles of 94°C 1 min, 52°C 0.5 min and 72°C for 1 min. PCR products cleaning and sequencing was performed either at Macrogen or at the Biotechnology Center DNA Sequencing Facility at the University of Wisconsin-Madison. The GenBank accession numbers for the entomopathogenic fungi isolates sequences are KY053448-KY053455 (ITS), KY055528-KY055532 (EF1-α) y KX579044-KX579053 (18S rRNA).

### Analysis of Sequences

Sequences from this study were edited using Geneious version 9.1.2 and high-quality sequences were clustered into operational taxonomic units (OTUs) using mothur (Schloss et al., 2009) with the standard divergence cut-off of 98% similarity (Schloss and Handelsman, 2004; Hong et al., 2006; Hulcr et al., 2011). Interactions bipartite network analysis were developed using the R package-bipartite software (Dormann et al., 2008).

Sequence alignments were performed using GUIDANCE (Penn et al., 2010) with MAFFT algorithm and 100 bootstrap repeats. Final editing of alignments was done in MEGA v6.0 (Tamura et al., 2013). Analyses for the best model of nucleotide substitution were performed using jModelTest2 version 2.1.6 (Darriba et al., 2012). The chosen model for 16S rRNA and *gyrB* sequences was GTR (General Time Reverse) and K80 (Kimura two-parameter) for ITS sequences. Phylogenetic analyses were performed through Bayesian inference, using MrBayes 3.2 (Ronquist and Huelsenbeck, 2003). All analyses employed one cold chain and three incrementally heated chains. A temperature parameter set to 0.2 for ribosomal RNA genes and 0.1 for *gyrB*. Four separate Markov Chain Monte Carlo runs were performed, with 3 million generations each (5 million generations for *gyrB*), discarding the initial 25% generations from each run as burn-in and sampling one in every 100 generations to calculate posterior probabilities for each branch. jModelTest and MrBayes analysis were carried out using the Cipress Science Gateway bioinformatics service platform (Miller et al., 2010). Final editing of each phylogenetic tree was done in FigTree v1.4 and Adobe Illustrator CS5.1.

### Bioassays

Duplicate bioassay challenges between Actinobacteria and entomopathogenic fungi were conducted. In each bioassay, both the bacteria and the fungi were isolated from the same insect host at the genus or species level. To obtain spore suspensions, fungal fermentations were done with a modified methodology (Suay et al., 2000). In the case of *Ophiocordyceps*/*Hirsutella* isolates, pieces of agar from pure cultures were transferred to 100 mL sterile Erlenmeyer’s flasks containing 20 mL of Grace Insect cell medium (Gibco) supplemented with 10% fetal bovine serum (Gibco). The inoculated flasks were shaken at 250 rpm at 25°C for 2–3 weeks. Spore suspensions were prepared by filtering the liquid culture through sterile cheesecloth placed in the tip of a sterile pipette while applying pressure with a pipette pump. Isolates classified in the genera *Metarhizium*, *Chlorocillium*, and *Engyodontium* were cultured directly in PDA plates for 3 weeks at 27°C. Spore suspensions were obtained by scrapping the mycelium directly from the agar media and transferring to 100 ml sterile Erlenmeyer flasks containing 20 mL of 0.1% tween-20 solution. Eight sterile crystal balls (3 mm diameter) were added to the flasks and then shaken at 250 rpm for 10 min. The mycelium was filtered through sterile cheesecloth and spore suspension collected in a sterile beaker. Suspension spore concentration was determined with a hemocytometer. Spore viability was assessed by inoculation of 100 μl spore suspension (∼1 × 10^6^ spore mL^-1^) on a PDA plate and incubation in the dark at 27°C and counting germinated spores within 24 h (Lacey, 2012).

PDA media was prepared and inoculated with the spore suspension to reach a final approximate concentration of 1 × 10^4^ viable spore mL^-1^. As we determined that temperatures above 40°C compromised spore viability of at least one of our fungal strains, the suspension spores were carefully preheated to 38°C and inoculated in PDA media at around 38–40°C. This avoid the formation of lumps when inoculating agar media with lower temperature spore suspension, keeping a homogenous mixture with viable spores. 24 mL PDA inoculated media was poured in Petri plate (8.5 cm in diameter). For each fungal strain, the panel of Actinobacteria isolates that corresponded to the same insect host were cultured in YMEA at 27°C for 2 weeks to reach the stationary phase growth. Then YMEA with Actinobacteria lawn growth was cut into 5 mm diameter disks and carefully transferred to the PDA media inoculated with entomopathogenic fungal spore suspension. Bioassays plates were incubated at 27°C for 2–4 weeks and halos of inhibition were measured. Negative controls were run with YMEA agar disks media only.

### Scanning Electron Microscopy

The ultrastructure of adults and nest material for two vespid wasps (*M. docilis* and *P. plebeja*) was examined. Two nest samples and adults per colony were fixed with glutaraldehyde and paraformaldehyde and were left at 4°C overnight. Samples were post-fixed in 1% osmium tetroxide for at least 1 h and dehydrated with ethanol (SEM; 30–100%) SEM and then dried by sublimation in a freeze dryer (VFD-20, VD Inc.). Due to the fragile nature of these samples, the processing was repeated with a modified Karnovsky and Osmium vapors fixation protocol (Kim, 2008; Alves et al., 2013). In this case, samples were put in a Petri plate containing a moistened cotton ball. In a fume hood, 1 mL of Karnovsky’s solution (Karnovsky, 1965) was placed in a small opened container that was then transferred into the Petri plate containing the sample. The Petri plate was kept closed and the sample was fixed at room temperature for 2 h. For a second fixation step, the container with Karnovsky solution was replaced with another container that contained 1 mL of 2% osmium tetraoxide. The Petri plate was covered with aluminum foil inside the fume hood and the sample was fixed for 12–24 h and then dried with silica gel in a hermetic container for 24 h. The samples were then mounted on aluminum stubs, with a double-stick carbon, coated with gold in a sputter Eiko ID 2 and examined under a scanning electron microscope Hitachi S-3700.

## Results

### Actinobacteria Isolation and Phylogenetic Analysis

In our sampling of 178 colonies of tropical hymenopteran insects, 197 isolates of Actinobacteria were obtained. Of the 29 different species of insects sampled, Actinobacteria was obtained from 22 (**Table 1**). Isolation of Actinobacteria from a particular species was only unsuccessful for species where sampling involved only a single colony, except in the case of the bee *Trigona* sp. 1 In comparing bees (34 colonies), ants (92 colonies), wasps (44 colonies), 23, 82, and 70 strains of Actinobacteria, were isolated respectively. By grouping the isolates according to the colony component from which they were obtained, 74 corresponded to adult insects (176 samples), 68 with immature insects (131 samples), and 45 with nest material (149 samples). In addition, from the stingless bee *T. angustula* other bee hive components were sampled and isolates obtained as follow: three from honey, one from brood comb, one from pollen and five from propolis (**Table 1**).

**FIGURE 1:**
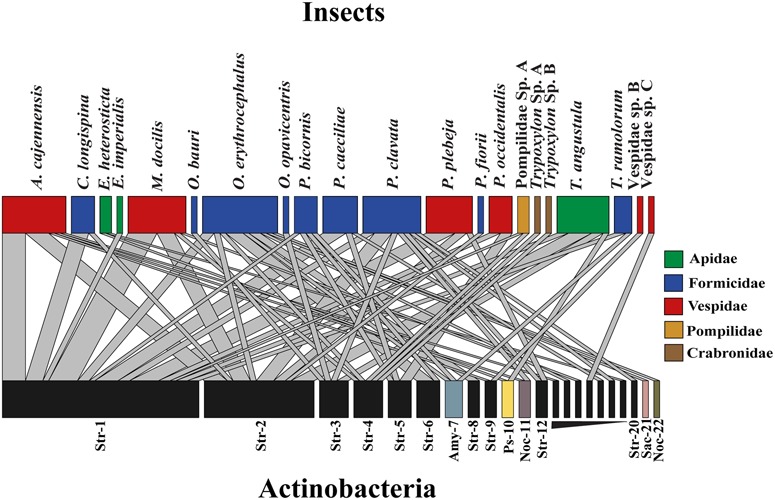
Insect-Actinobacteria interaction network using OTUs clustering with 2% cut-off sequence similarity. Upper level boxes represent insect species and lower level boxes depict associated Actinobacteria OTUs. Boxes and link size are proportional to the total number of isolates obtained and to the frequency of this particular interaction, respectively. Genera are abbreviated as follow: *Streptomyces* (Str), *Pseudonocardia* (Ps) *Amycolatopsis* (Amy) *Nocardia* (Noc) *Saccharothrix* (Sac).

From the 197 Actinobacteria-like isolates obtained, we choose only unique morphotypes per colony component sampled (174), for DNA extraction and 16S rRNA sequence analysis. From those isolates 161 successfully amplified, 23 were replicates, and 138 Actinobacteria sequences were chosen for further analysis. A sequence alignment was generated and then 44 short sequences were removed and the remaining 94 were clustered into 22 different OTUs with a 2% cutoff used as an indicator of bacterial species (**Figure 1**, see Supplementary Table S1 for details about the distribution of isolates differentiating their origin) (Hong et al., 2006; Hulcr et al., 2011). Interaction network analysis using OTUs clustering did not show evidence of specific associations between Actinobacteria isolates and the insect species from which they were obtained (**Figure 1**). *Streptomyces* isolates were grouped into 22 different OTUs with ten of them being represented by singletons (**Figure 1**). Six dominant *Streptomyces* OTUs accounted for 75% of all sequences included in the analysis. Two *Pseudonocardia* strains associated with bees and three *Amycolatopsis* strains associated with ants were also found. Within the Actinobacteria genus *Nocardia*, one strain was isolated from a bullet ant (*P. clavata*) and wasp larvae *(A. cajennensis*) and a different strain obtained from nest material of a trap-jaw ant colony (*O. erythrocephalus*). Additionally, one *Sacchrothrix* strain was isolated from *O. erythrocephalus* larvae (**Figure 1**).

**FIGURE 2:**
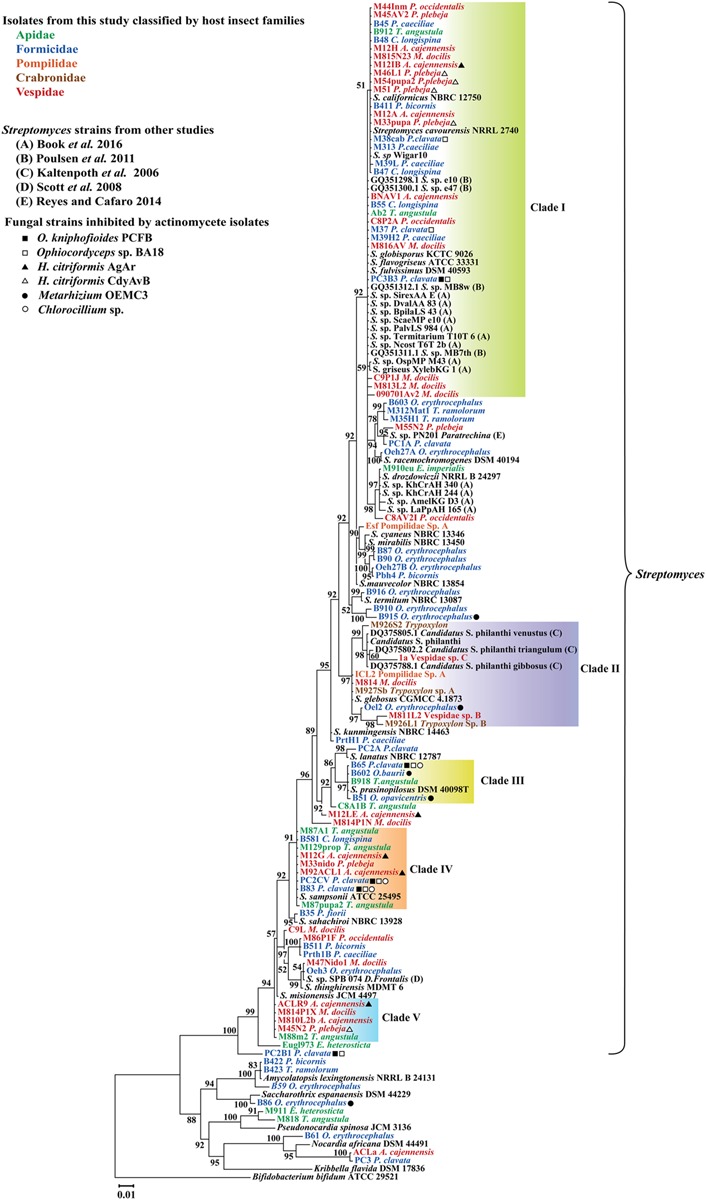
Bayesian, phylogenetic analysis of 16S rRNA sequences of Actinobacteria associated with different hymenopteran insect species. Insect taxonomic identity is indicated next to sample code. Posterior probabilities (PP) are indicated at the corresponding nodes. NCBI reference sequences included in the analysis are denoted by the corresponding type culture collection acronym. Clades within 99% sequence similarity cutoff are highlighted except for clade II that it is composed of different phylotypes and was highlighted to show a group of sequences closely related to the well-studied solitary wasp symbiont *Candidatus* Streptomyces philanthi. Alignment 950 pb.

**FIGURE 3:**
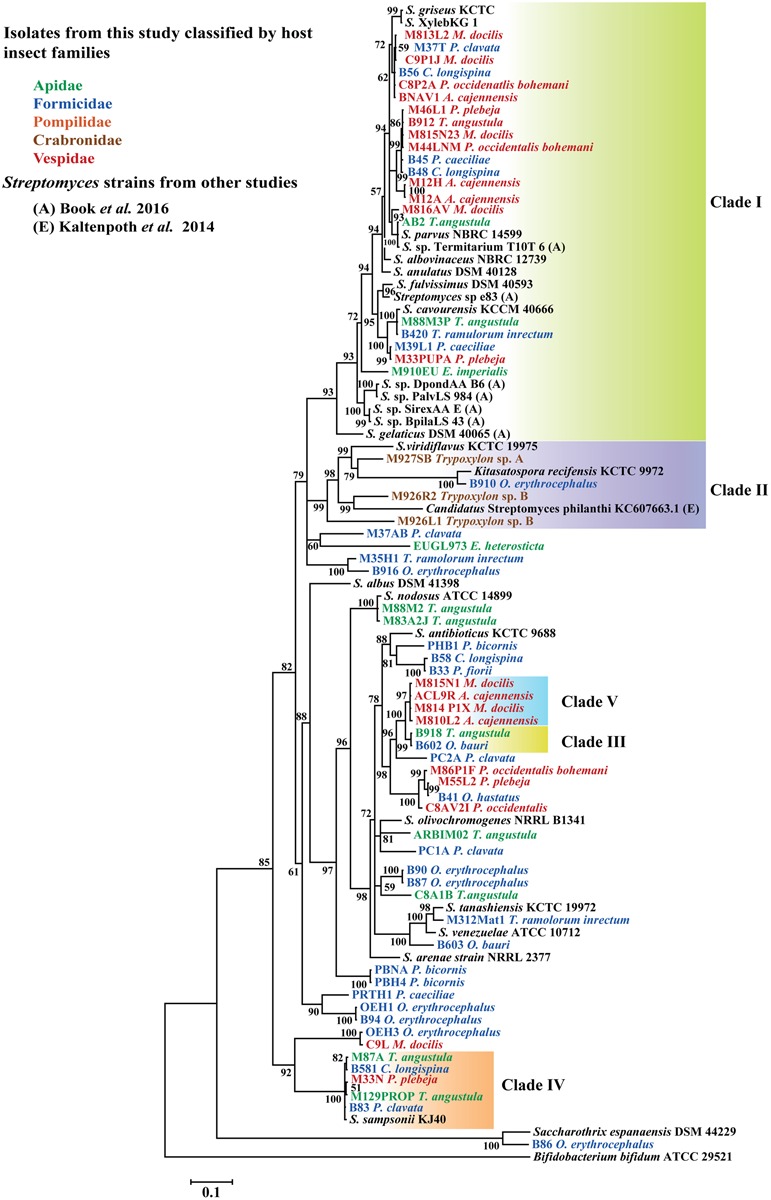
Bayesian, phylogenetic analysis of partial *gyrB* sequences of *Streptomyces* associated with different hymenopteran insect species. Insect taxonomic identity is indicated next to sample code. Posterior probabilities (PP) are indicated at the corresponding nodes. NCBI reference sequences included in the analysis are denoted by the corresponding type culture collection acronym. Representative isolates with 99% sequence similarity cutoff in the 16S rRNA phylogeny are highlighted as well as clade II that it is composed of different phylotypes. Clade II was highlighted to show a group of sequences closely related to the well-studied solitary wasp symbiont *Candidatus* Streptomyces philanthi. Alignment 780 pb.

Four main clades of Actinobacteria, each one composed of sequences with 99% 16S rRNA identity were found to be associated with different groups of insects under study (**Figure 2**). In general, phylogenetic analysis of *gyrB*, grouped representative sequences from the 16S rRNA tree in the same clades (**Figures 2, 3**). In the 16S rRNA phylogenetic tree, the paraphyletic group I represents a single 16S rRNA phylotype isolated repeatedly from 29 different samples, from nine different hymenopteran insect species (**Figure 2**). This group contained 16 *Streptomyces* isolates associated with vespid wasp colonies, 11 from ants and 2 from the stingless bee *T. angustula*. The closest relatives to this group were *S. fulvissimus* DSM 43, *S. flavogriseus* ATCC 33331, *S. globisporus* KTC 9026 and *S. cavourensis* NRRL 2740, based on >99% sequence similarity. Moreover, sequences from other studies corresponding to known host associated *Streptomyces* strains with antimicrobial (Poulsen et al., 2011) or cellulolytic activity (Book et al., 2016) were clustered in clade I (**Figure 2**). In the *gyrB* phylogeny, representative sequences within clade I were resolved into different taxonomic groups but within a single clade with the *Streptomyces* strains with high cellulolytic activity (**Figure 3**).

Clades III, IV, and V also represent different 16S rRNA phylotypes (99% cut off) with their closest relatives being *S. prasinopilosus*, *S. sampsonii*, and *S. misionensis*, respectively. Sequences corresponding to *Streptomyces* isolates associated with subsocial Crabronidae or Pompilidae insects grouped together in the monophyletic Clade II (**Figure 3**) with *Candidatus* Streptomyces philanthi, the solitary wasp *Philanthus triangulum* symbiont (Kaltenpoth et al., 2005, 2014).

### Electron Microscopy for Actinobacteria Localization in Brood Chambers and Insect’s Cuticle

**FIGURE 4:**
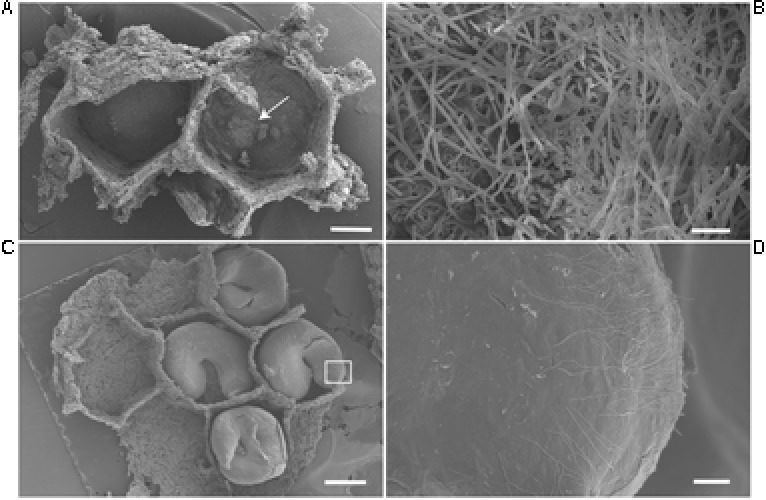
SEM image of the interior of wasp brood chambers. **(A)** Two brood chambers of *M. docilis*, after larvae removal. Scale, 1 mm. **(B)** Filamentous bacteria forming a dense cluster at the base of the chamber, hyphae diameters of 1.3 – 2.4 μm. Scale, 20 μm. **(C)** *P. plebeja* larvae in brood chambers. Scale, 1 mm. **(D)** *Streptomyces* like hyphae growing on *P. plebeja* larvae. Hyphae diameter of 1.2 – 1.7 μm. Scale, 60 μm.

From the 24 Actinobacteria isolates associated with the wasps *M. docilis*, 62% were obtained from the nest substrate. Scanning electronic micrographs from brood chambers from two different *M. docilis* nests revealed the presence of filamentous microorganisms (**Figure 4B**). Vegetative hyphae with diameters of 1.3 – 2.4 μm was abundant at the base of the brood chamber but was not observed externally (**Figures 4A,B**). In the case of the vespid wasp *P. plebeja*, six different colonies were studied from which were obtained 11 *Streptomyces* isolates associated with brood or nest samples. Four of these isolates were isolated from different colonies and grouped together as one 16S rRNA *Streptomyces* phylotype in Clade I (**Figure 2**), suggesting being a common strain associated with this wasp. The SEM ultrastructure of *P. plebeja* brood chambers was also studied and bacteria with *Streptomyces* morphology were found growing on the larvae (**Figures 4C,D**). These hyphae have diameters between 1.2 and 1.7 μm. The ultrastructure of adult *M. docilis* wasp cuticle was also studied; however, evidence of microorganism growth was not found. Similarly, no hyphae with morphology similar to *Streptomyces* were seen when samples from adults, immature stages, and nest material from the vespid *A. cajennensis* and adult ants within the species *O. erythrocephalus* and *P. clavata* were seen under SEM.

### Isolation and Identification of Entomopathogenic Fungi

**FIGURE 5:**
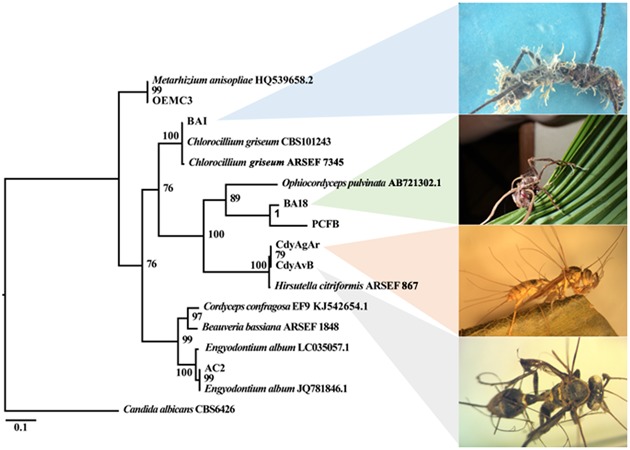
Bayesian phylogenetic relationships among fungal isolates in this study as inferred from ITS-5.8S sequences. Posterior probability (PP) at the corresponding nodes. The tree was rooted on *Candida albicans.*

The ITS region from seven fungal isolates that were obtained from insect cadavers was successfully amplified and a phylogenetic tree was constructed (**Figure 5**). These isolates grouped with the families Ophiocordycipitaceae, Metacordycipitacea, and other closely related groups.

**FIGURE 6:**
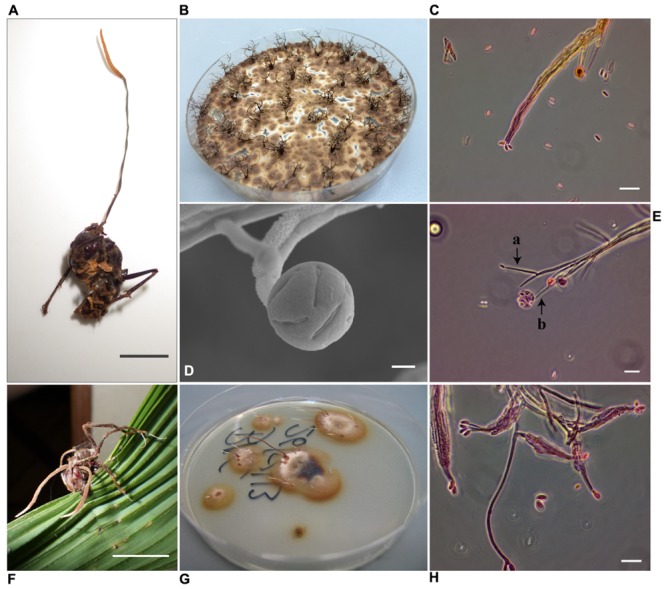
*Ophiocordyceps* fungi infecting *P. clavata* ants. **(A)** *Ophiocordyceps kniphofioides* (*H. stilbelliformis*) PCFB in the sexual state. Scale, 1 cm **(B)** *H. stilbelliformis* PCFB isolate pure culture on PDA, showing dense growth of synnemata. **(C)** *H. stilbelliformis* PCFB ovoid conidia borne at compacted terminal phialides. Scale, 20 μm **(D)** SEM micrograph of PCFB verrucose apical phialide with conidias inside mucous droplet. Scale, 4 μm. **(E)** a- and b- phialides and conidia of *Hirsutella stilbelliformis* PCFB. Scale, 20 μm. **(F)** *P. clavata* ant, infected by *Ophiocordyceps* sp. Scale, 1 cm. **(G)** Isolate BA18 in PDA pure culture. **(H)** BA18, verrucose compact and apical phialides with ovoid conidia. Scale, 20 μm.

PCFB and BA18, two fungal strains isolated from *P. clavata* ants were classified by macro and micromorphology as well as ITS, SSU and E-1α sequences, within the Ophiocodycipitaceae family where all *Cordyceps* fungi infecting ants are grouped. Isolate PCFB was obtained from a specimen with fungal growth in its teleomorph stage, with a characteristic single brown stroma with orange fertile tip emerging from the back of the ant’s head (**Figure 6A**). This isolate was identified as *O. kniphofioides* (*H. stilbelliformis*) with a 98% Ef-1α sequence identity. Moreover, microscopic study of coordinated hyphal structures (synnemata) of isolate PCFB, showed the presence of distinctive verrucose hyphae, phialides, and conidia (**Figures 6B–E**) (Evans and Samson, 1984). Isolate BA18 (**Figure 6G**) was obtained from a *P. clavata* ant attached by its mandibles to the underside of a palm leaf (**Figure 6F**) and the fungus was growing out of the ant’s body in its anamorphic state. Isolate BA18 was recognized as a close relative to *O. kniphofioides* sharing 95% Ef-1α and 98% 18S partial sequence similarity when compared against the closest relatives sequence available at NCBI database. Conidiophores and conidia structures similar to isolate PCFB were found when the micromorphology of BA18 synnemata (**Figure 6H**) was examined. A *Metarhizium* strain isolated from an *O. erythrocephalus* ant, was included in our entomopathogen collection. This asymptomatic specimen was collected in the field and brought to the lab and later died developing *Metarhizium* like fungal growth. The identity of this fungus was confirmed by ITS sequence.

**FIGURE 7:**
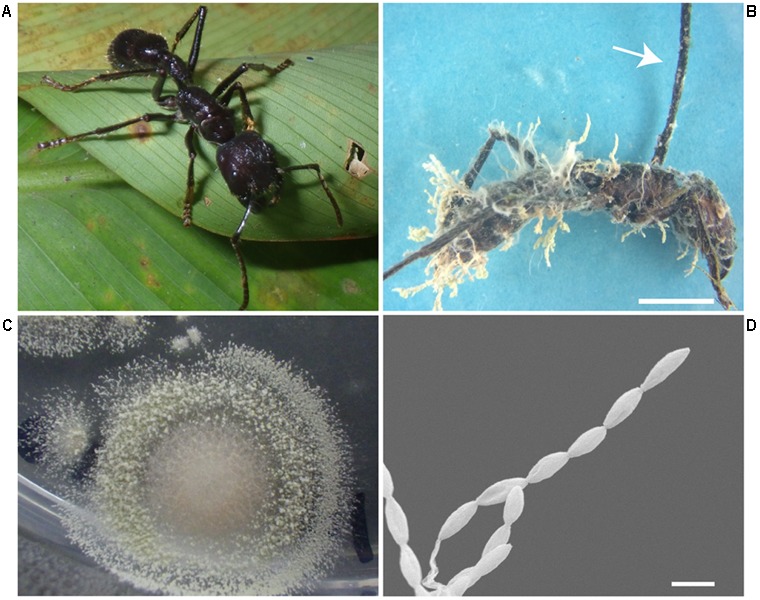
*Paraponera clavata* ant parasitized by fungi. **(A)** Healthy *P. clavata* ant. **(B)** Infected ant, arrow pointing at the stroma that grew on the back of the ant’s head with no perithecia, note the dense yellowish aerial hyphae with chains of conidia growing on the ant’s body. Scale, 5 mm **(C)** Isolate BAI obtained from the *P. clavata* cadaver. **(D)** SEM micrograph, showing the conidia chains growing directly from the ant’s infected body. Scale, 2 μm.

**FIGURE 8:**
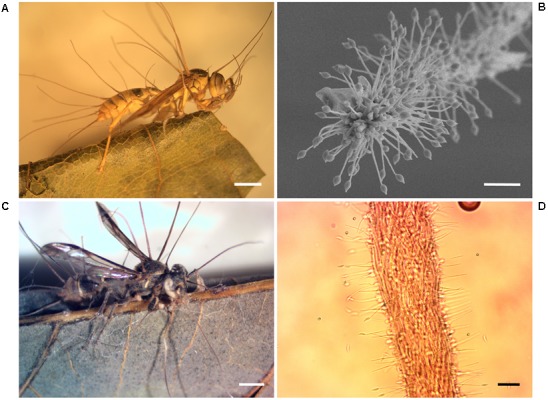
Wasps infected with *Hirsutella* fungi. **(A)** *A. areata* cadaver. Note solitary stromata, filiform, arising from thorax to the abdomen region of the host, erect or curved and of brown color. Scale, 2 mm. **(B)** Ultrastructure of stromata from isolate CdyAgAr showing phialides and citriform conidia. Scale, 20 μm. **(C)** *Polybia* sp. wasp attached to a leaf by their mandibles. Scale, 1 mm. **(D)** Light micrograph of stromata from CdyAVB isolate in PDA pure culture. Scale, 20 μm.

BAI was an isolate obtained from an apparently hyperparasitized *P. clavata* specimen presenting a brown dried stroma stalk extending from the back of the ant’s head and a dense yellowish aerial mycelium with spores growing out from the joints of the cadaver (**Figure 7**). Isolate BAI was grouped together with *Chlorocillium* spp. by its ITS and 18S gene sequencing. Isolates CdyAgAr and CdyAvB, obtained from wasp specimens collected at San Carlos were identified as *Hirsutella* sp. (**Figure 8**). For these isolates, ITS and 18S rRNA genes were sequenced, and both genes shared 99% identity with *H. citriformis* when compared against sequences from NCBI databases. Sequence similarity, as well as macro and microscopic morphology examination, suggest that both wasp specimens were infected by the same fungal species. Finally, a fungal isolate classified by ITS and SSU sequences as *Engyodontium* sp. was included in the study. This isolate was obtained from an *Agelaia* wasp cadaver infected by an *Ophiocordyceps* fungus that was collected at La Selva in November 2014. Although, to our knowledge there is no evidence of *Engyodontium* fungus being pathogens of hymenopteran insects, there have been reports of this fungal genus being a pathogen of another insect group (Samson et al., 2013).

### Bioassays: Actinobacteria Strains with Antifungal Properties against Insects’ Natural Enemies of Fungal Origin

**FIGURE 9:**
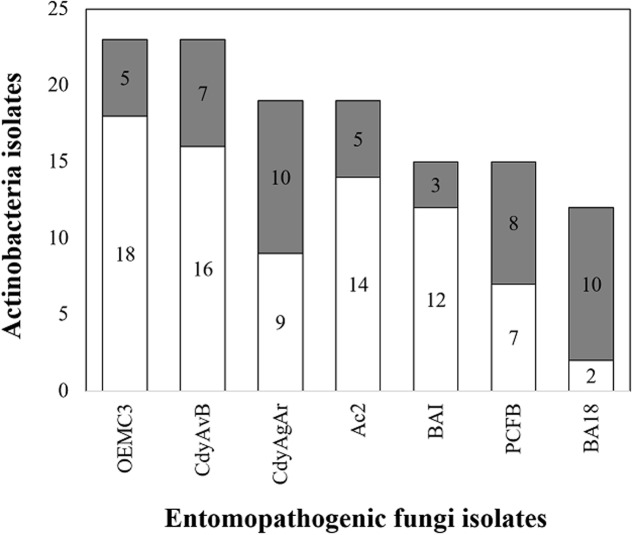
Actinobacteria isolates with antifungal properties against entomopathogenic fungi. In gray, the number of isolates with antifungal properties are shown and in white the ones without activity. OEMC3 (*Metarhizium* isolate against Actinobacteria obtained from *Odontomachus* ants), CdyAgAR (*H. citriformis* isolate against Actinobacteria obtained from *A. cajennensis*), CdyAvB (*H. citriformis* against Actinobacteria isolated form *P. occidentalis* y *P. plebeja* wasps), AC2 (*Engyodontium* sp. against Actinobacteria associated with *A. cajennensis*), BAI (*Chlorocillium* sp. challenged against *P. clavata* Actinobacteria isolates), PCFB (*O. kniphofioides* against *P. clavata* Actinobacteria) y BA18 (*Ophiocordyceps* sp. against *P. clavata* Actinobacteria).

In total 125 bioassays were carried out in duplicates between Actinobacteria and entomopathogenic fungi. Only some of the Actinobacteria isolates were used in bioassays and were chosen so that both the bacteria and the fungi were obtained from the same insect host, at the species or genus level. These bioassays were categorized into seven different groups each one corresponding to a different fungus. For every group of bioassays, at least three different Actinobacteria isolates that could inhibit the growth of the pathogenic fungi *in vitro* (**Figure 9**) were found. The highest proportion of antifungal activity was seen for bioassays between *P. clavata* Actinobacteria and the fungal isolate BA18. In this group of bioassays, 10 out 12 Actinobacteria isolates inhibited the growth of the *Ophiocordyceps* fungus. In the case of *P. clavata* Actinobacteria and the PCFB (*O. kniphofioides*) fungal isolate, 8 out 15 bacterial isolates produced halos of inhibition. Similarly, in *A. cajennensis* and *Hirsutella* bioassays, 12 out of 23 isolates presented antifungal activity.

All Actinobacteria isolates with antifungal activity that had their 16S rRNA sequence included in the phylogenetic study were mapped in the tree (**Figure 2**). From the four Actinobacteria isolates not belonging to the genus *Streptomyces* that were screened for antifungal properties, only B86 (identified in the genus *Saccharothrix*) was effective in inhibiting the growth of the fungal strain that was tested against (*Metarhizium*).

## Discussion

Our results indicate that Actinobacteria associations with hymenopteran insects are more common than previously described (Currie et al., 1999; Kaltenpoth et al., 2005; Promnuan et al., 2009; Poulsen et al., 2011; Madden et al., 2013; Reyes and Cafaro, 2014). With SEM analysis, the presence of filamentous microorganisms with cell morphology similar to *Streptomyces* inside *M. docilis* and *P. plebeja* brood chambers (**Figure 4**) were confirmed. Moreover, Actinobacteria from most insect species sampled were successfully isolated, including insects from five different Hymenopteran families, which represent eusocial (paper wasps, ants, and the stingless bee, *T. angustula*) and non-eusocial lifestyles (orchid bees, mud dauber wasps in the family Crabronidae and a spider wasp in the family Pompilidae). The highest proportion of isolates per colony was obtained in *Trypoxylon* sp. wasps, followed by paper wasps in the tribe Epiponini (*M. docilis*, *P. plebeja*, *P. occidentalis*, and *A. cajennensis*). In these cases, Actinobacteria were isolated from all colony compartments sampled (brood, adults and nest material). This was also the case for ant species such as *O. erythrocephalus*, *C. longispina*, *P. caeciliae*, and *T. ramulorum inrectum*. On the other hand, with the exception of the meliponid bee *Trigona* sp. 1 (four colonies sampled), all insect species that did not yield Actinobacteria were sampled only once. In the case of *Trigona* bees, only adults and material from nest entrances were sampled, as the colonies studied were located inside tree trunk cavities. It is possible that no Actinobacteria were isolated from *Trigona* bees because of the inability to sample other colony components. It has been suggested that the role of defensive Actinobacteria symbionts could be linked to specific vulnerable life stages of their host (e.g., larvae and pupae; Kaltenpoth and Engl, 2013). Likewise, in a different study, Promnuan et al. (2009) reported the isolation of Actinobacteria from brood cells of *Trigona* bee hives. Consequently, we obtained 12 actinomycete isolates from the internal colony components of a related stingless bee, *T. angustula.*

*Streptomyces* was the predominant genus of culturable Actinobacteria associated with insect colonies in the present study. These results are consistent with similar studies employing culture-dependent isolation approaches in *Apis* and *Trigona* bees (Promnuan et al., 2009), *Dendroctonus* beetles (Hulcr et al., 2011), *Polistes dominulus* wasps (Madden et al., 2013) and *Pseudomyrmex penetrator*, *Petalomyrmex phylax*, and *Crematogaster margaritae* ants as well as culture-independent techniques, as the case for *P. longicornis* (Reyes and Cafaro, 2014). Moreover, our interaction network analysis suggests that the dominant *Streptomyces* phylotypes had diverse associations with hymenopteran hosts at both the genus and family levels (**Figure 1**). The binning of isolates from different insect groups into a few OTUs imply that possible horizontal transmission and selective processes may have occurred. These results agree with other well-studied insect-Actinobacteria defensive symbiosis in which the symbiont has undergone dynamic substitution over the evolutionary history of their host (Kaltenpoth et al., 2010, 2012; Cafaro et al., 2011; Kaltenpoth and Engl, 2013). Although some Actinobacteria strains classified into genera different than *Streptomyces* were isolated, our culture-dependent screening approach may have underestimated the insect host Actinobacteria diversity. Independent cultivation techniques are necessary to address the real diversity, abundance and stability of Actinobacteria in these systems.

Our results indicate that 1/3 of all the isolates obtained in our sampling occur within clade I of our 16S rRNA phylogenetic tree. This clade corresponds to a lineage of highly cellulolytic *Streptomyces* previously identified in temperate zones, which is associated with a variety of insect species that feed on plant biomass (Book et al., 2016). The high cellulolytic activity present in these bacteria appears to be the result of evolutionary adaptations acquired through horizontal transfer and selective retention of genes as well as the expansion of their regulatory elements (Book et al., 2016). Further studies evaluating the cellulolytic activity and genomic content enrichment for key enzyme families related to plant biomass degradation in our isolates from clade I would provide important insight into this potential nutritional role of these Actinobacteria in their host niche.

To test the resolution of our 16S rRNA tree, the *gyrB* gene from representative isolates included in our 16S rRNA phylogeny were amplified and sequenced. Our 16S rRNA and *gyrB* phylogenies had corresponding sequences grouped in the same clades with minor changes related to more variability in sequence composition in clade I from the *gyrB* tree (**Figure 3**). As pointed out elsewhere, among important housekeeping genes markers, *gyrB* produced higher correlations with genome relatedness in phylogenetic analysis with Actinobacteria belonging to the Streptomycetaceae family (Han et al., 2012). When comparing the phylogenetic trees, there is evidence of apparently unique phylotypes represented in clades III, IV, and V (**Figures 2, 3**) in both phylogenies. These monophyletic clades consist of one taxonomic unit that was isolated repeatedly from different insect hosts. Moreover, isolates within these clades that were included in bioassays showed antifungal properties against different pathogenic fungi, suggesting that these isolates shared similarities in terms of production of antifungal compounds active against natural enemies of their host.

Our phylogenetic analysis results also suggest that subsocial wasps included in our study and the *Streptomyces* isolates obtained from them are taxonomically related to *P. triangulum* and its mutualistic symbiont *Ca.* Streptomyces philanthi respectively, showing congruency in both Actinobacteria-insect association systems. These findings require further study, as the *P. triangulum*-*Streptomyces* defensive symbiosis is thought to be confined into the subfamily Philanthinae (Kaltenpoth et al., 2012), whereas *Trypoxylon* is a genus belonging to the subfamily Crabroninae. Both of these subfamilies are classified in different monophyletic clades in the diverse paraphyletic family Crabronidae (Debevec et al., 2012).

One important goal of this study was to test if the Actinobacteria associated with insects can protect them against natural enemies. Different strains of *Ophiocordyceps* (*Hirsutella*) fungi were isolated and blastospores were obtained in high concentrations to screen for Actinobacteria antifungal activity *in vitro*. All of the insect species examined (those from which entomopathogenic fungi were isolated) had more than three different associated Actinobacteria strains that were capable of producing zones of inhibition in co-culture *in vitro* bioassays (**Figure 9**).

**FIGURE 10:**
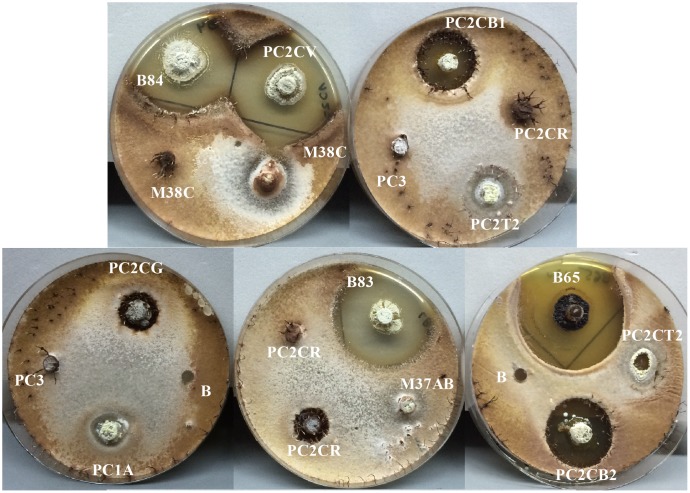
Bioassay, antifungal activity screening from Actinobacteria isolates associated with *P. clavata* against *O. kniphofioides*. The Actinobacteria isolate codes are shown. Negative controls are marked with the letter “B.” For illustrative purposes the photographs were taken after 4 weeks of incubation because of the slow growing of this fungus.

*Cordyceps* fungi are characterized by being host specialized parasites. For example, the zombie ant fungus *Ophiocordyceps unilateralis* is an antagonist of ants in the tribe Camponotini (Formicinae, Formicidae), with less common infections reported on *Polyrhachis* ants (Pontoppidan et al., 2009; Andersen et al., 2012). Similarly, *Ophiocordyceps kniphofioides* infects *Cephalotes atratus* (Formicidae: Myrmicinae), *Paraponera clavata*, and *Dinoponera longipes* (Formicidae: Ponerinae) ants (Evans and Samson, 1982; Sanjuan et al., 2015). In this study, it has been shown that different strains of *Streptomyces* isolated from *P. clavata* ants could inhibit the ant’s parasitic fungus *O. kniphofioides* (**Figure 10**).

Two *H. citriformis* (Ophiocordycipitaceae: Hypocreales) strains were isolated from two different vespid wasps (**Figure 8**). *Hirsutella* is a genus of anamorphic fungi whose teleomorphs has commonly been linked to the genus *Ophiocordyceps* (Sung et al., 2007). *H. citriformis* is a known pathogen of insects in the orders Hemiptera and Psocoptera (Simmons et al., 2015). To our knowledge, this is the first report on *H. citriformis* infecting Hymenopteran insects. In this study, *H. citriformis* was also inhibited by several wasp associated Actinobacteria strains. Our results suggest that the antifungal substances produced by symbiotic Actinobacteria may occur in the natural environment, but additional studies are necessary to demonstrate their presence.

This study shows associations of a few *Streptomyces* lineages with diverse neotropical hymenopteran insects, one of them being previously described in temperate zones, suggesting a consistent association that spans from temperate to tropical regions. Moreover, the results of the present study provide evidence of antagonism between some Actinobacteria strains and the fungal entomopathogens tested and supports the idea that certain insects may use some of the active secondary metabolites from associated Actinobacteria as an additional protection mechanism against natural enemies. Collectively, these findings provide insight into the widespread and dynamic host-niche selection of Actinobacteria and points to promising models of mutualistic symbiosis, which harbor great potential in finding new biologically active compounds with medical and biotechnological applications.

## Author Contributions

Conceived and designed the experiments: CC, AP-T, MM, and BM-C. Performed the experiments: BM-C. Processed SEM samples: RM-S. Analyzed the data: BM-C, CC, and AP-T. Contributed reagents/materials/analysis tools: CM-C and MM. Wrote the paper: BM-C, CC, and AP-T.

## Conflict of Interest Statement

The authors declare that the research was conducted in the absence of any commercial or financial relationships that could be construed as a potential conflict of interest.
