# Supplementary material for: Evidence for Widespread Associations between Neotropical Hymenopteran Insects and Actinobacteria
Source: Front Microbiol. 2017 Oct 17;8:2016. doi: 10.3389/fmicb.2017.02016 (PMC5651009; doi:10.3389/fmicb.2017.02016)
Supplement: Supplementary file 1 [file Table_1.docx]

Supplementary Material

**Evidence for Widespread Associations Between Neotropical Hymenopteran Insects and Actinobacteria.**

Bernal Matarrita-Carranza^1, 2^, Rolando D. Moreira-Soto^2, 3^, Catalina Murillo-Cruz^2^, Marielos Mora^4^, Cameron R. Currie^5^, Adrián A. Pinto-Tomas^2, 4, 6^

*** Correspondence:** adrian.pinto@ucr.ac.cr

# Supplementary Tables

Table S1. Description of the Actinobacteria isolates obtained by insect colony sampled.

| **Colony** | **Insect host** | **Colony component** | **Isolate code** | **OTUs *** |
| --- | --- | --- | --- | --- |
| BM13060701 | *Agelaia cajennensis* | adult | BNAV1 | Str-1 |
| RV12111803 | *Agelaia cajennensis* | inmature insect | M12A | Str-1 |
| RV12111803 | *Agelaia cajennensis* | nest | M12H | Str-1 |
| RV12111804 | *Agelaia cajennensis* | inmature insect | M12IB | Str-1 |
| RV11040901 | *Crematogaster longispina* | inmature insect | B48 | Str-1 |
| RV11040901 | *Crematogaster longispina* | adult | B47 | Str-1 |
| RV11050703 | *Crematogaster longispina* | adult | B55 | Str-1 |
| RV12090802 | *Euglossa imperialis* | adult | M910Eu | Str-1 |
| RV12082604 | *Metapolybia docilis* | inmature insect | M813L2 | Str-1 |
| RV12083101 | *Metapolybia docilis* | nest | M815N23 | Str-1 |
| RV12083102 | *Metapolybia docilis* | adult | M816AV | Str-1 |
| RV12090701 | *Metapolybia docilis* | nest | C9P1J | Str-1 |
| RV12090701 | *Metapolybia docilis* | adult | 090701av2 | Str-1 |
| BM13012701 | *Odontomachus erythrocephalus* | adult | Oeh27A | Str-1 |
| RV11060401 | *Odontomachus erythrocephalus* | nest | b603 | Str-1 |
| BM15070303 | *Paraponera clavata* | adult | PC3B3 | Str-1 |
| RV12031607 | *Paraponera clavata* | adult | M37T | Str-1 |
| RV12031608 | *Paraponera clavata* | adult | M38 cab | Str-1 |
| RV11041602 | *Paratrechina caeciliae* | adult | B45 | Str-1 |
| RV12032401 | *Paratrechina caeciliae* | adult | M39H2 | Str-1 |
| RV12032401 | *Paratrechina caeciliae* | inmature insect | M39L1 | Str-1 |
| RV12032405 | *Paratrechina caeciliae* | inmature insect | M313Inm | Str-1 |
| RV11041606 | *Pheidole bicornis* | inmature insect | B411 | Str-1 |
| RV12031603 | *Polybia plebeja* | inmature insect | M33pupa | Str-1 |
| RV12040105 | *Polybia plebeja* | adult | M45AV2 | Str-1 |
| RV12041301 | *Polybia plebeja* | inmature insect | M46L1 | Str-1 |
| RV12041306 | *Polybia plebeja* | inmature insect | M51 | Str-1 |
| RV12041403 | *Polybia plebeja* | inmature insect | M54 pupa2 | Str-1 |
| RV12082502 | *Polybia occidenatlis bohemani* | nest | C8p2A | Str-1 |
| RV12040104 | *Polybia occidenatlis bohemani* | Inmature insect | M44Inm | Str-1 |
| RV12090805 | *Pompilidae* | adult | ESF | Str-1 |
| RV12031605 | *Tapinoma ramolorum* | adult | M35H1 | Str-1 |
| RV12032404 | *Tapinoma ramolorum* | nest | M312 mat1 | Str-1 |
| BM11091103 | *Tetragonisca angustula* | Propoleo | B912 | Str-1 |
| RV12082503 | *Tetragonisca angustula* | adult | Ab2 | Str-1 |
| RV12082601 | *Agelaia cajennensis* | inmature insect | M810L2 | Str-2 |
| RV12090102 | *Agelaia cajennensis* | inmature insect | M92ACL1 | Str-2 |
| RV12111801 | *Agelaia cajennensis* | nest | M12G | Str-2 |
| RV13092704 | *Agelaia cajennensis* | inmature insect | ACL9R | Str-2 |
| RV11050804 | *Crematogaster longispina* | inmature insect | B581 | Str-2 |
| RV12041302 | *Metapolybia docilis* | nest | M47Nido1 | Str-2 |
| RV12082605 | *Metapolybia docilis* | nest | M814P1X | Str-2 |
| RV12090701 | *Metapolybia docilis* | inmature insect | C9L | Str-2 |
| BM13072701 | *Odontomachus erythrocephalus* | adult | Oeh3 | Str-2 |
| BM15070302 | *Paraponera clavata* | adult | PC2CV | Str-2 |
| RV11080602 | *Paraponera clavata* | adult | B83 | Str-2 |
| RV11030407 | *Pheidole fiorii* | adult | B35 | Str-2 |
| RV12031603 | *Polybia plebeja* | nest | M33 nido | Str-2 |
| RV12040105 | *Polybia plebeja* | nest | M45N2 | Str-2 |
| RV12082503 | *Tetragonisca angustula* | inmature insect | M87pupa2 | Str-2 |
| RV12082503 | *Tetragonisca angustula* | adult | M87A1 | Str-2 |
| RV12082504 | *Tetragonisca angustula* | miel | M88m2 | Str-2 |
| RV12113001 | *Tetragonisca angustula* | Propoleo | M129prop | Str-2 |
| RV11061003 | *Odontomachus bauri* | inmature insect | B602 | Str-3 |
| RV11050706 | *Odontomachus opavicentris* | inmature insect | B51 | Str-3 |
| RV11060305 | *Paraponera clavata* | adult | B65 | Str-3 |
| RV11090802 | *Tetragonisca angustula* | adult | B918 | Str-3 |
| RV12081102 | *Tetragonisca angustula* | adult | C8A1B | Str-3 |
| RV12082605 | *Metapolybia docilis* | nest | M814 Pm | Str-4 |
| BM13072101 | *Odontomachus erythrocephalus* | inmature insect | OEL2 | Str-4 |
| RV12090805 | *Pompilidae* | inmature insect | ICL2 | Str-4 |
| RV12092205 | *Trypoxylon sp a* | adult | M927Sb | Str-4 |
| RV12092204 | *Trypoxylon sp B* | adult | M926S2 | Str-4 |
| RV13062901 | *Paratrechina caeciliae* | adult | PrtH1b | Str-5 |
| RV11050702 | *Pheidole bicornis* | nest | B511 | Str-5 |
| RV12082502 | *Polybia occidenatlis bohemani* | nest | M86P1F | Str-5 |
| RV12082505 | *Polybia occidenatlis bohemani* | adult | C8AV2I | Str-5 |
| BM13072701 | *Odontomachus erythrocephalus* | adult | Oeh27B | Str-6 |
| RV11080601 | *Odontomachus erythrocephalus* | adult | B87 | Str-6 |
| RV11060302 | *Odontomachus erythrocephalus* | adult | B90 | Str-6 |
| RV13062903 | *Pheidole bicornis* | nest | PBh4 | Str-6 |
| RV11052701 | *Odontomachus erythrocephalus* | inmature insect | B59 | Amy-7 |
| RV11040905 | *Pheidole bicornis* | nest | B422 | Amy-7 |
| RV11040908 | *Tapinoma ramolorum* | adult | B423 b | Amy-7 |
| BM11091102 | *Odontomachus erythrocephalus* | adult | B910 | Str-8 |
| RV11090801 | *Odontomachus erythrocephalus* | adult | B915 | Str-8 |
| Rv12092204 | *Trypoxylon sp B* | inmature insect | M926L1 | Str-9 |
| RV12082602 | *Vespidae sp B* | inmature insect | M811L2 | Str-9 |
| RV12090803 | *Euglossa heterosticta* | adult | M911 | Ps-10 |
| RV12081101 | *Tetragonisca angustula* | Peine | M818 | Ps-10 |
| BM13060701 | *Agelaia cajennensis* | inmature insect | ACLa | Str-11 |
| BM15070303 | *Paraponera clavata* | adult | PC3 | Noc-11 |
| BM13060701 | *Agelaia cajennensis* | Inmature insect | ACLA | Noc-11 |
| RV12041404 | *Polybia plebeja* | nest | M55 n2 | Str-12 |
| BM13101201 | *Paraponera clavata* | adulto | PC1A | Str-12 |
| RV12111805 | *Agelaia cajennensis* | inmature insect | M12LE | Str-13 |
| BM11091102 | *Odontomachus erythrocephalus* | adult | B916 | Str-14 |
| RV12082605 | *Metapolybia docilis* | nest | M814p1N | Str-15 |
| RV12090704 | *Vespidae sp C* | inmature insect | 1A | Str-16 |
| RV13062901 | *Paratrechina caeciliae* | adult | PrtH1 | Str-17 |
| BM13101202 | *Paraponera clavata* | adult | PC2A | Str-18 |
| RV12090703 | *Euglossa heterosticta* | adult | EUGL973 | Str-19 |
| BM15070302 | *Paraponera clavata* | adult | PC2B1 | Str-20 |
| RV11080601 | *Odontomachus erythrocephalus* | inmature insect | B86 | Sac-1 |
| RV11060402 | *Odontomachus erythrocephalus* | nest | B61 | Noc-22 |

* Actinobacteria genera are abbreviated as follow: Streptomyces (Str), Pseudonocardia (Ps) Amycolaptosis (Amy) *Nocardia* (Noc) *Saccharothrix* (Sac).
